# Identification of Nontuberculous Mycobacteria in Patients with Pulmonary Diseases in Gyeongnam, Korea, Using Multiplex PCR and Multigene Sequence-Based Analysis

**DOI:** 10.1155/2021/8844306

**Published:** 2021-02-22

**Authors:** Min-Jeong Kim, Kyu-Min Kim, Jeong-Ih Shin, Jong-Hun Ha, Dong-Hae Lee, Jeong-Gyu Choi, Jin-Sik Park, Jung-Hyun Byun, Jung-Wan Yoo, Seokyong Eum, Myunghwan Jung, Seung Chul Baik, Woo Kon Lee, Hyung Lyun Kang, Min-Kyoung Shin

**Affiliations:** ^1^Department of Microbiology, College of Medicine, Gyeongsang National University, Jinju 52727, Republic of Korea; ^2^Department of Convergence Medical Sciences, Institute of Health Sciences, Gyeongsang National University, Jinju 52727, Republic of Korea; ^3^Department of Laboratory Medicine, Gyeongsang National University Hospital, Jinju 52727, Republic of Korea; ^4^Department of Internal Medicine, Gyeongsang National University Hospital, Jinju 52727, Republic of Korea; ^5^International Tuberculosis Research Center, Changwon 51755, Republic of Korea

## Abstract

**Background:**

Nontuberculous mycobacteria (NTM) are widely present in environments, such as soil and water, and have recently been recognized as important pathogenic bacteria. The incidence of NTM-related infections is steadily increasing. As the diagnosis and treatment of NTM infection should be distinguished from tuberculosis, and the treatment should be specific to the species of NTM acquired, accurate species identification is required.

**Methods:**

In this study, two-step multiplex PCR (mPCR) and multigene sequence-based analysis were used to accurately identify NTM species in 320 clinical isolates from Gyeongsang National University Hospital (GNUH). In particular, major mycobacterial strains with a high isolation frequency as well as coinfections with multiple species were diagnosed through two-step mPCR. Multigene sequencing was performed to accurately identify other NTM species not detected by mPCR. Variable regions of the genes 16S rRNA, *rpoB*, *hsp65*, and 16S-23S rRNA internal transcribed spacer were included in the analysis.

**Results:**

Two-step mPCR identified 234 (73.1%) cases of *M*. *intracellulare*, 26 (8.1%) cases of *M*. *avium* subsp. *avium*, and 13 (4.1%) cases of *M*. *avium* subsp. *hominissuis* infection. Additionally, 9 (2.8%) *M*. *fortuitum*, 9 (2.8%) *M*. *massiliense*, 2 (0.6%) *M*. *abscessus*, and 4 (1.2%) *M*. *kansasii* isolates were identified. Coinfection was identified in 7 (2.2%) samples. The sixteen samples not classified by two-step mPCR included 6 (1.9%) cases of *M*. *chimaera*, 4 (1.3%) *M*. *gordonae*, 1 (0.3%) *M*. *colombiense*, 1 (0.3%) *M*. *mageritense*, and 1 (0.3%) *M*. *persicum* identified by sequence analysis.

**Conclusions:**

The results of this study suggest a strategy for rapid detection and accurate identification of species using two-step mPCR and multigene sequence-based analysis. To the best of our knowledge, this study is the first to report the identification of NTM species isolated from patients in Gyeongnam/Korea.

## 1. Introduction

Nontuberculous mycobacteria (NTM) include mycobacterial species other than the members of the *Mycobacterium tuberculosis* (MTB) complex and *M*. *leprae* [1, 2]. More than 200 NTM species have been identified [3]. Human-to-human transmission of NTM has not yet been demonstrated; therefore, NTM infections are generally considered to be environmentally acquired [4]. NTM are widely present in the environment, such as in soil and water; thus, humans can be infected by NTM through regular activities [5]. Recent studies have investigated the relationship between *M*. *avium* complex (MAC) strains isolated from the patients' residential areas and those isolated from the patients themselves. It has been reported that a higher number of strains has been isolated from patients' residences than from public places [6, 7]. Additionally, NTM outbreaks may be associated with contaminated hospital and commercial water supplies [8, 9]. Moreover, a recent study has described a prolonged outbreak of *M*. *chimaera* associated with a heater cooling device used in cardiopulmonary surgery [10]. This shows that devices used in medical and research facilities can be a source of NTM infections and that the occurrence of this type of NTM infection is likely to continue [10, 11]. NTM have emerged as important human pathogens [12]. The development of new methods that improve NTM identification is therefore needed.

Global surveillance shows that the incidence of MTB infections is decreasing, but that of NTM infections has rapidly increased since the 1990s [13]. Unlike MTB infections, public health authorities do not monitor NTM infections; moreover, even though the prevalence of NTM infection is steadily increasing, its incidence may be underestimated [4, 12, 14]. NTM infections frequently cause lung diseases, which account for more than 90% of diseases due to NTM; other diseases due to NTM include lymphadenitis, skin disease, and disseminated disease [15, 16]. The species that cause lung diseases mainly include MAC, *M*. *abscessus*, *M*. *kansasii*, and *M*. *chelonae* [17]. NTM are generally resistant to primary antituberculosis drugs and require a different set of treatments from MTB because its progression of infection and disease characteristics are completely unique [14]. Furthermore, because infectious NTM species differ in their resistance to treatments and outcomes, the identification of NTM species is clinically important [14, 18]. The correct identification of NTM species depends on the molecular databases that have been built to date, and protocols for NTM-specific diagnosis are still developing and require improvement [14]. Further studies on developing better diagnostic tools and treatments for diseases due to NTM are required [19]. Currently, NTM infections are diagnosed based on the diagnostic standards of the American Thoracic Society and the American Society of Infectious Diseases, which include standards for species that commonly cause NTM lung diseases such as *M*. *kansasii* but not for all NTM [20].In this study, a fast and accurate mycobacterial identification strategy was developed using multiplex PCR (mPCR) and multigene sequence-based analyses [21]. First, two-step mPCR was developed to identify the causative agents of major mycobacterial infections: MTB and major NTM, *M*. *intracellulare*, and *M*. *avium* in the first mPCR and *M*. *kansasii*, *M*. *fortuitum*, *M*. *abscessus*, and *M*. *massiliense* in the second mPCR. Unidentified mycobacteria in mPCR were then identified through 16S ribosomal RNA (16S rRNA), RNA polymerase *β* subunit (*rpoB*), heat shock protein 65 (*hsp65*), and 16S-23S rRNA internal transcribed spacer (ITS) sequence-based analysis [19, 21–26]. Two-step mPCR was performed for 320 NTM clinical isolates from patients with pulmonary diseases treated at the Gyeongsang National University Hospital between 2016 and 2018. The major MTB complex, *M*. *avium*, and *M*. *intracellulare* could be identified in the first mPCR reaction and *M*. *kansasii*, *M*. *fortuitum*, *M*. *abscessus*, and *M*. *massiliense* in the second mPCR reaction. Mycobacteria not identified in the two-step mPCR were identified using a multigene sequence-based analysis.

## 2. Materials and Methods

### 2.1. Bacterial Strains

A total of 320 NTM clinical isolates from patients with pulmonary diseases treated at the Gyeongsang National University Hospital (GNUH; Jinju, Korea) between 2016 and 2018 were used in this study. Additionally, nine reference strains, MTB (NCCP 72077), *M*. *bovis* (BCG Pasteur 1173P2), *M*. *avium* subsp. *hominissuis* 104, *M*. *avium* subsp. *avium* (KBN12P06234), *M*. *intracellulare* 9514 (ATCC 13950), *M*. *abscessus* (KCTC 19621), *M*. *massiliense* (KCTC 19086), *M*. *fortuitum* (KBN12P06244), and *M*. *kansasii* (KBN12P06233) were used to validate the two-step mPCR method developed in the present study.

### 2.2. Bacterial Culture and DNA Extraction

Mycobacteria were cultured on 7H10 agar (BD Difco, USA) containing albumin-dextrose-saline (10×:50 g/L BSA, 20 g/L dextrose, 8.5 g/L NaCl) and incubated at 37°C for 7–14 days. Genomic DNA was extracted using a PrimePrep Genomic DNA Extraction Kit from Tissue (GeNetBio, Korea) according to the manufacturer's instructions.

### 2.3. Two-Step mPCR Amplification

Genomic DNA from each sample was used to perform the two-step mPCR, with a total of nine primer sets. The primer sequences used are listed in Table 1. For the first mPCR reaction, the template DNA was completely denatured at 95°C for 10 min, followed by 30 cycles of denaturation at 95°C for 30 s, annealing at 61°C for 40 s, extension at 72°C for 1 min, and finally an additional extension at 72°C for 10 min. The reaction mixtures contained the DNA template (1 ng/*μ*L), forward and reverse primers, 1× reaction buffer (0.15 mM MgCl_2_), dNTP mix (25 *μ*M each tube), and 1 U Taq DNA polymerase (Bioneer, Korea). Sterilized water was added to obtain a final volume of 20 *μ*L per tube. IS*901* primers were used at a concentration of 1 pM/*μ*L, whereas 16S rRNA, rv0577, RD9, IS*311*, and DT1 primers were used at a concentration of 0.3 pM/*μ*L.

For the second mPCR reaction, the template DNA was completely denatured at 95°C for 10 min, followed by 30 cycles of denaturation at 95°C for 30 s, annealing at 62°C for 40 s, extension at 72°C for 1 min, and an additional extension at 72°C for 10 min. Primer pairs, such as transferase, *mass_3210*, and SOD, were used at a concentration of 0.3 pM/*μ*L. The composition of the reaction mixture, excluding the primers, was the same as that of the first mPCR reaction. The PCR products for each amplification were separated using a 1.5% Tris-acetate-EDTA (TAE) agarose gel and 1× TAE buffer and then analyzed and visualized with a UV transmittal illuminator.

### 2.4. Multigene Sequence-Based Analysis

Unidentified clinical isolates from the two-step mPCR were assessed using multigene sequencing. Genomic sequences of reference strains were obtained from the National Center for Biotechnology Information (NCBI). Primer pairs were designed in conserved regions flanking the most variable regions after sequence alignment using CLUSTAL_X version 2.1 [28]. The four primer sets used for multigene sequencing are shown in Table 1. For amplification and sequencing of 16S rRNA and *rpoB*, the amplification was performed for 5 min at 95°C followed by 30 cycles at 95°C for 30 s, 60°C for 40 s, and 72°C for 1 min, with a final extension at 72°C for 10 min. Reaction mixtures contained the DNA template (1 ng/*μ*L), forward and reverse primers (1 pM/*μ*L), 1× reaction buffer (0.15 mM MgCl_2_), dNTP mix (25 *μ*M each tube), and 1 U Taq DNA polymerase (Bioneer). Sterilized water was added to obtain a final volume of 20 *μ*L per tube. For the amplification and sequencing of *sp65*, the PCR conditions were 5 min at 95°C, followed by 30 cycles of 95°C for 30 s, 61°C for 40 s, and 72°C for 1 min, with a final extension at 72°C for 10 min. The reaction mixtures contained the DNA template (10 ng/*μ*L), forward and reverse primers (1 pM/*μ*L), 1× reaction buffer (0.15 mM MgCl_2_), dNTP mix (25 *μ*M each tube), and 1 U Taq DNA polymerase (Bioneer). Sterilized water was added to obtain a final volume of 20 *μ*L per tube. Multigene sequence similarity for the clinical isolates was determined in comparison with the reference sequences in the multigene database using the basic local alignment search tool (BLAST).

### 2.5. Phylogenetic Analyses

Multigene sequence alignment was performed using the CLUSTAL X version 2.1 tool [28]. Phylogenetic trees were constructed based on multigene sequences using the MEGA_X program [29] and four different neighbor-joining methods with the maximum composite likelihood method. Bootstrap analysis (1000 repeats) was performed to evaluate the topology of each phylogenetic tree.

## 3. Results

### 3.1. Development of Two-Step mPCR Using Clinical Isolates

**Figure 1:**
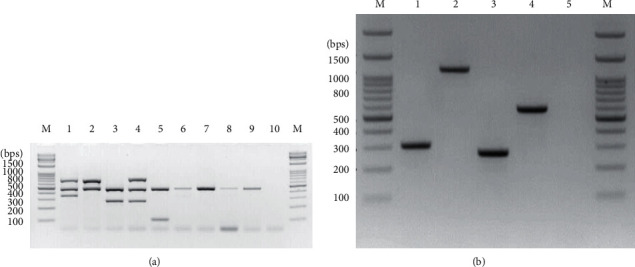
Validation of the two-step multiplex PCR using reference strains. (a) The first mPCR reaction was conducted with mycobacterial reference strains. The amplified products were of the following sizes: Lane M, DNA ladder; Lane 1, 358 bps (RD9), 484 bps (16s rRNA), and 705 bps (rv0577) for MTB NCCP 72077; Lane 2, 484 bps (16s rRNA) and 705 bps (rv0577) for *M*. *bovis* BCG Pasteur 1173P2; Lane 3, 282 bps *(IS1311)* and 484 bps (16s rRNA) for *M*. *avium* subsp. *hominissuis* 104; Lane 4, 282 bps *(IS1311)*, 484 bps (16s rRNA), and 753 bps *(IS901)* for *M*. *avium* subsp. *avium* KBN12P06234; Lane 5, 106 bps (DT1) and 484 bps (16s rRNA) for *M*. *intracellulare* ATCC 13950; Lane 6, 484 bps (16s rRNA) for *M*. *kansasii* KBN12P06233; Lane 7, *M*. *abscessus* KCTC 19621; Lane 8, *M*. *massiliense* KCTC 19086; Lane 9, *M*. *fortuitum* KBN12P06244; and Lane 10, negative control. (b) The second mPCR reaction was conducted with mycobacterial reference strains. The amplified products were of the following sizes: Lane M, DNA ladder; Lane 1, 310 bps (*mass_3210*) for *M*. *abscessus*; Lane 2, 1145 bps (*mass_3210*) for *M*. *massiliense*; Lane 3, 275 bps (SOD) for *M*. *fortuitum*; Lane 4, 582 bps (transferase) for *M*. *kansasii*; and Lane 5, negative control.

The two-step mPCR method was performed using nine reference strains. The mPCR products were readily visualized on an agarose gel. In the first mPCR reaction, the bands were 358 bps (RD9), 484 bps (16s rRNA), and 705 bps (rv0577) for MTB; 484 bps (16s rRNA) and 705 bps (rv0577) for *M*. *bovis*; 282 bps (IS*1311*) and 484 bps (16s rRNA) for *M*. *avium* subsp. *hominissuis*; 282 bps (IS*1311*), 484 bps (16s rRNA), and 753 bps (IS*901*) for *M*. *avium* subsp. *avium*; 106 bps (DT1) and 484 bps (16s rRNA) for *M*. *intracellulare*; and 484 bps (16 s rRNA) for *M*. *kansasii*, *M*. *abscessus*, *M*. *massiliense*, and *M*. *fortuitum* (Figure 1(a)). In the second mPCR, the bands were 310 bps (*mass_3210*) for *M*. *abscessus*; 1145 bps (*mass_3210*) for *M*. *massiliense*; 275 bps (SOD) for *M*. *fortuitum*; and 582 bps (transferase) for *M*. *kansasii* (Figure 1(b)).

**Figure 2:**
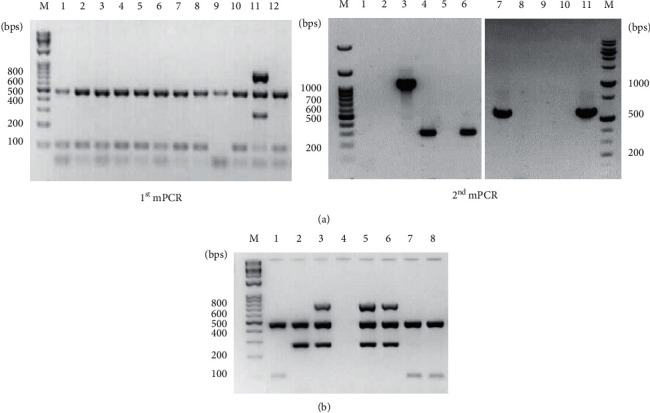
Representative results of mPCR using clinical isolates and detection of coinfection. A total of 320 clinical isolates were tested for the identification of mycobacterial species using the two-step mPCR method. (a) Results of the first and second mPCR using clinical isolates. The clinical isolates were analyzed using the first mPCR with *M*. *intracellulare* (Lanes 1–8, 10, and 12) and *M*. *avium* subsp. *avium* (Lane 11). The strain with only 16sRNA (484 bps, Lane 9) bands was later identified through the secondary mPCR or multigene sequence analysis. In particular, Lane 11, with the *IS901* (753 bps), 16sRNA (484 bps), *IS1311* (282 bps), and DT1 (106 bps) bands, was suspected as a *M*. *avium* subsp. *avium* or *M*. *avium* subsp. *avium* and *M*. *intracellulare* coinfection (the first mPCR, left panel). The clinical isolates were identified as *M*. *abscessus* (Lane 1), *M*. *massiliense* (Lane 2), and *M*. *kansasii* (Lane 3) (second mPCR, right panel). (b) Confirmation of coinfection results: *M*. *avium* subsp. *avium* (Lanes 5–6) and *M*. *intracellulare* (Lanes 7–8) were single-isolated and identified from a suspected coinfection sample in Lane 11 of panel A Lane M, DNA ladder; Lane 2, *M*. *avium* subsp. *hominissuis* 104; Lane 3, *M*. *avium* subsp. *avium* KBN12P06234; Lane 4, negative control.

From 2016 to 2018, 320 patients with NTM infections were treated at GNUH and subjected to this diagnostic strategy (Figure 2(a)). In the first mPCR reaction, 234 (73.1%) isolates were identified as *M*. *intracellulare*, 26 (8.1%) as *M*. *avium* subsp. *avium*, and 13 (4.1%) as *M*. *avium* subsp. *hominissuis*. In the second mPCR reaction, 9 (2.8%) isolates were identified as *M*. *fortuitum*, 9 (2.8%) as *M*. *massiliense*, 2 (0.6%) as *M*. *abscessus*, and 4 (1.2%) as *M*. *kansasii*. Sixteen samples that could not be classified using the two-step mPCR were assessed through multigene sequencing of the 16S rRNA, *rpoB*, *hsp65*, and ITS for accurate identification of the NTM species. Six (1.9%) cases of *M*. *chimaera* infection, four (1.3%) *M*. *gordonae* infection, one (0.3%) *M*. *colombiense* infection, one (0.3%) *M*. *mageritense* infection, and one (0.3%) *M*. *persicum* infection were identified via sequence analysis (Table 2).

Using this molecular diagnostic method, seven clinical samples showed mixed mPCR results for two or more species and multiple NTM coinfections were suspected. As shown in Lane 11 of Figure 2(b), the 106 bps (DT1), 282 bps (IS*1311*), 484 bps (16sRNA), and 753 bps (IS*901*) signals were simultaneously identified, indicating possible coinfection with *M*. *avium* subsp. *avium* and *M*. *intracellulare*. Thus, to confirm the presence of coinfection, the sample was subjected to a single isolation, and the first mPCR was performed. Further, *M*. *avium* subsp. *avium* (Lanes 5–6) and *M*. *intracellulare* (Lanes 7–8) were successfully isolated, and coinfection with *M*. *avium* subsp. *avium* and *M*. *intracellulare* was confirmed (Figure 2(b)).

Coinfection was suspected in seven (2.2%) of the total samples (Table 2); three of these with the IS*901*, 16s rRNA, IS*1311*, and DT1 bands were suspected with *M*. *avium* subsp. *avium* and *M*. *intracellulare* coinfection and one with IS*311*, 16s rRNA, and DT1 bands was suspected with *M*. *avium* subsp. *hominissuis* and *M*. *intracellulare* coinfection. One case with DT1 and 16s rRNA bands in the first mPCR showed a transferase band in the second mPCR, confirming a coinfection with *M*. *intracellulare* and *M*. *kansasii*. In addition, one case showed RD9, 16s rRNA, and rv0577 bands in the first mPCR and mass_3210 (1145 bp) bands in the second mPCR, indicating MTB and *M*. *massiliense* coinfection and one case with DT1, RD9, 16s rRNA, and rv0577 bands was suspected of MTB and *M*. *intracellulare* coinfection (data not shown).

### 3.2. Determination of Mycobacterial Partial Gene Target for Multigene Sequence-Based Analysis

Target genes such as 16S rRNA, *rpoB*, *hsp65*, and ITS were selected for mycobacterial identification, and their sequences were analyzed to identify the NTM species that were not identified using the two-step mPCR. Using the gene sequence information of 16S rRNA, *rpoB*, and *hsp65* registered in GenBank for 17 NTM reference species strains, the conserved and variable regions of each gene target were classified, and the critical partial variable regions were determined for comparison with whole gene sequences.

The 16S rRNA gene, which is approximately 1500 bp, was determined as a target for the multigene sequencing assay with a variable region at 140–620 bp. Furthermore, 17 NTM species were compared, and the results are presented in Table 3. The homology of the partial 16S rRNA sequence among the 17 reference strains was 83.6–100%. The homology of *M*. *bovis*, *M*. *tuberculosis*, *M*. *abscessus*, *M*. *chelonae*, *M*. *chimaera*, *M*. *indicus*, and *M*. *intracellulare* was >99.6%, which was indistinguishable using partial 16S rRNA sequences. However, among species other than these highly homologous ones, the identity of partial 16S rRNA was lower than that of the full 16S rRNA sequence, making it possible to distinguish between different NTM species more clearly (Supplementary Table 1).

The *rpoB* gene has a size of approximately 3500 bp, and the variable region at 291–1255 bp was determined as a target for the multigene sequencing assay. The identities of the 17 NTM species are presented in Supplementary Table 2. The similarity of the partial *rpoB* sequences among the 17 reference strains was 84.8–100%. In addition, *M*. *bovis* and *M*. *tuberculosis* could not be accurately distinguished as the homology of their *rpoB* sequences was 100%. Furthermore, *M*. *abscessus* and *M*. *chelonae* had 95.2% identity, which was more clearly distinguishable from their respective 16S rRNAs. The similarity between *M*. *chimaera* and *M*. *intracellulare* was <99.7%, and the similarity between *M*. *colombiense* and *M*. *chimaera* and *M*. *intracellulare* was 95.9%, which was clearly distinguishable.

*Hsp65* has a size of approximately 1600 bp, and the variable region at 620–1220 bp was determined as a target for the multigene-sequencing assay. Supplementary Table 3 shows a comparison of *hsp65* identity among the 17 NTM species. The identity of the partial *hsp65* sequence among the 17 reference strains was 89–100%. The similarity between *M*. *bovis* and *M*. *tuberculosis* and that between *M*. *chimaera* and *M*. *intracellulare* were 100%, but other species were clearly distinguishable.

ITS is a region between the 16S rRNA gene and the 23S rRNA gene, and because its size varies across species, its sequence can be easily analyzed [26]. A forward primer was designed at the downstream of the 16S rRNA gene and a reverse primer in the upstream of the 23S rRNA gene; thus, the full ITS sequence was used for the analysis (Supplementary Figure 1). The identity of the full ITS sequence among the 17 reference strains was 62.7–100%. Although it was still impossible to distinguish between species with 100% homology, such as *M*. *bovis* and *M*. *tuberculosis*, the other NTM strains were clearly distinguishable.

### 3.3. Identification of Clinical Isolates by Multigene Sequence-Based Analysis

The 16 clinical isolates that were unidentified through the two-step mPCR were analyzed using BLAST in multigene sequence-based assays. Table 3 shows the results of a multigene sequence-based analysis comparing the four target gene sequences of the 16 clinical isolates with the sequences of the reference strains and those obtained from the NCBI database. Six clinical strains were identified as *M*. *chimaera* (CI-1, 5, 6, 8, 11, and 13), all of which showed homology in 16S rRNA (99.3–100%), *rpoB* (99%–100%), *hsp65* (99.5–100%), and ITS (98%–100%). In addition, four clinical strains were identified as *M*. *gordonae* (CI-2, 3, 7, and 12), showing homology in 16S rRNA (99.8, 100%), *rpoB* (99.9, 100%), *hsp65* (98.1, 100%), and ITS (98.5, 100%). The clinical isolate identified as *M*. *colombiense* (CI-4) showed 100% similarity in 16S rRNA, 96.6% in *rpoB*, 99% in *hsp65*, and 98.4% in ITS. In addition, the isolates identified as *M*. *persicum* (CI-9) and *M*. *mageritense* (CI-10) showed 99.2% and 99.6% similarity in 16S rRNA, 99.8% and 99.8% in *rpoB*, 99.1% and 97.6% in *hsp65*, and 90.9% and 94% in ITS, respectively. However, the three clinical isolates ND#1, ND#2, and ND#3, which were not clearly identified through multigene sequence-based analysis, were subjected to phylogenetic analysis using multigene sequences.

### 3.4. Phylogenetic Analyses

**Figure 3:**
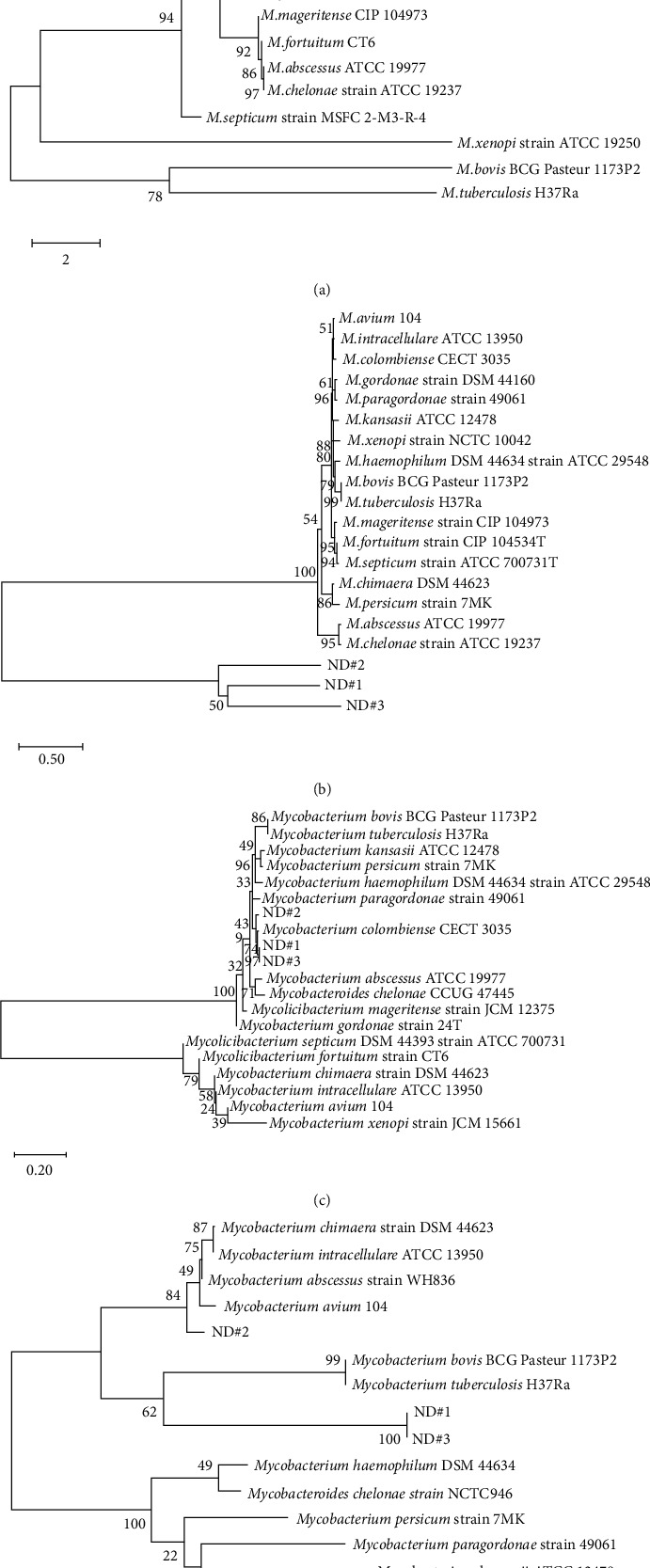
Phylogenetic trees based on the 16S rRNA (a), *rpoB* (b), *hsp65* (c), and ITS (d) sequences of the six ND strains and mycobacterial reference strains. Bootstrap values (expressed as a percentage of 1,000 replications) are given at corresponding branch nodes.

Four phylogenetic trees were constructed based on partial 16S rRNA, *rpoB*, and *hsp65* sequences and full ITS sequences from the three clinical isolates unassigned to a known species from multigene sequencing. In the 16S rRNA sequence-based trees, the three clinical isolates, ND#1, ND#2, and ND#3, were clearly grouped together with strains derived from the MAC, *M*. *kansasii*, and *M*. *haemophilum* (Figure 3(a)). In the rpoB sequence-based trees, ND#1, ND#2, and ND#3 were highly similar to one another but were separated from other strains and were out-grouped (Figure 3(b)). In the *hsp65* sequence-based trees, ND#1, ND#2, and ND#3 were highly similar to *M*. *colombiense* (Figure 3(c)). ND#1 and ND#3, which were highly similar to each other, showed similarity to the MTB complex, whereas ND#2 showed high similarity to MAC (Figure 3(d)).

## 4. Discussion

Bacterial culture and phenotyping are traditional NTM identification methods. However, it is still necessary to identify isolates to obtain information on antibiotic resistance because NTM are resistant to primary antituberculosis drugs. To avoid unnecessary treatment delays, a molecular diagnostic method for the identification of mycobacterial species has been developed [30]. Molecular identification of mycobacterial species has advantages in terms of speed, accuracy, reproducibility, and the ability to identify mycobacteria directly from clinical samples [30]. In recent years, the incidence of NTM infections in patients hospitalized in Korea has increased. Thus, among the infections caused by mycobacteria, MTB and NTM infections should be immediately distinguished and the NTM species that caused the disease should be clearly and accurately identified.

As a molecular diagnostic method for identifying mycobacterial species, mPCR assays that are relatively fast, accurate, and easy to apply have been continuously developed. In particular, the present study aimed to develop the two-step mPCR assay as a new diagnostic strategy using a modified mPCR technique based on the results of previous studies [21, 22, 27]. Shin et al. developed an mPCR technique that distinguishes subspecies within MAC [21], whereas Chae et al. focused on the diagnosis of *tuberculosis* to the extent of distinguishing even the Beijing type of MTB in MTBC and identifying NTM species including MAC, *M*. *abscessus*, *M*. *massiliense*, and *M*. *kansasii* through mPCR [22]. Singh et al. identified major NTM species, including MAC, *M*. *abscessus*, and *M*. *kansasii*, using the mPCR technique but could not distinguish between *M*. *avium* and *M*. *intracellulare* in MAC [31].

In this study, a two-step mPCR assay was used to identify the major NTM and MTB species common in both Korea and abroad. This method quickly and accurately diagnosed major mycobacterial species in two mPCR steps. MTB and major NTM, *M*. *intracellulare* and *M*. *avium*, were identified in the first mPCR, and *M*. *kansasii*, *M*. *fortuitum*, *M*. *abscessus*, and *M*. *massiliense* were identified in the second mPCR. In this study, 320 clinical isolates were obtained from patients treated at the Gyeongsang National University Hospital. In the first mPCR reaction, *M*. *intracellulare* was the most common species, identified in 234 (73.1%) isolates, followed by *M*. *avium* subsp. *avium* (26, 8.1%) and *M*. *avium* subsp. *hominissuis* (13, 4.1%). In the second mPCR reaction, *M*. *fortuitum* was isolated in 9 (2.8%) samples, *M*. *massiliense* in 9 (2.8%), *M*. *abscessus* in 2 (0.6%), and *M*. *kansasii* in 4 (1.3%). Coinfection was observed in seven (2.2%) samples. These cases of coinfection included three *M*. *avium* subsp. *avium* and *M*. *intracellulare*; one *M*. *avium* subsp. *hominissuis* and *M*. *intracellulare*; one *M*. *intracellulare* and *M*. *kansasii*; one MTB and *M*. *intracellulare*; and one MTB and *M*. *massiliense*. Altogether, more than 95% of NTM strains could be identified using this method. When multiple bands were detected in the first and second mPCR developed in this study, coinfection could be confirmed. However, the results could not determine coinfection with other strains of the same species or coinfection with mycobacteria that were not detected by this mPCR assay.

Other NTMs that were not identified by the two-step mPCR were identified by multigene sequencing using specific gene targets, such as 16s rRNA, *rpoB*, *hsp65*, and ITS. For accurate identification, sequencing analysis of the full sequence of each gene should be performed [26]. However, 16S rRNA, *rpoB*, and *hsp65* have sizes of approximately 1500, 3500, and 1600 bp, respectively, which are too large for accurate sequencing analysis. Therefore, partial regions were selected within these genes to perform sequencing analyses quickly and accurately. 16S rRNA sequencing is widely used for bacterial research, and when a new species is identified, its information is registered with the NCBI database. However, in NTM, there is little variation between species; thus, to identify NTM species, 16S rRNA sequencing alone may be insufficient. In this study, the homology among *M*. *abscessus*, *M*. *chelonae*, *M*. *tuberculosis*, and *M*. *bovis* was 100% in 16S rRNA, but these four species could be distinguished by mPCR. The homology of MAC complexes, such as *M*. *chimaera*, *M*. *colombiense*, and *M*. *intracellulare,* was more than 99.6%, making it impossible to distinguish the species using 16S rRNA sequences alone. However, the homologies of the partial sequences were lower than the homology of the entire sequence in other NTM species (Supplementary Table 1). In the case of the *hsp65* gene, there were many variable regions, similar to the *rpoB* gene; thus, it could be easily identified in different species [22, 26, 32]. However, there were many unreliable data on rapidly growing bacteria when only using the BLAST search. In the *rpoB* gene, *M*. *bovis* and *M*. *tuberculosis* were indistinguishable because they showed 100% homology, but it was possible to distinguish *M*. *abscessus* and *M*. *chelonae* that showed 95.2% homology, which would otherwise be indistinguishable through 16S rRNA sequencing. Among the MAC complexes, *M*. *intracellulare* and *M*. *chimaera* were difficult to distinguish because of 99.5% homology and *M*. *intracellulare* and *M*. *indicus* because of 99.7% homology, but the remaining MAC complexes could be distinguished (Supplementary Table 2). The *hsp65* gene exhibits a homology tendency similar to that of *rpoB*. The homology among *M*. *intracellulare*, *M*. *chimaera*, and *M*. *indicus* was greater than 99.6%, making them indistinguishable, but that among other MAC complexes was distinct (Supplementary Table 3). The ITS region is easy to assess because of its variability across different species. The size of the ITS DNA fragments between species was also distinguishable. Among the MAC complexes, it was not possible to distinguish between *M*. *intracellulare* and *M*. *indicus* because of their 100% homology, but the remaining MAC complexes could be clearly distinguished (Supplementary Figure 1). Using this multigene sequence-based analysis, the 16 unidentified clinical isolates were identified, and 6 (1.9%) were found to be *M*. *chimaera*, 4 (1.3%) *M*. *gordonae*, 1 (0.3%) *M*. *colombiense*, 1 (0.3%) *M*. *mageritense*, 1 (0.3%) *M*. *persicum*, and 3 (0.9%) ND strains. As with the mPCR technique, studies on the identification of NTM species using multigenes such as 16s rRNA, *rpoB*, *hsp65*, and ITS have been conducted previously. Davari et al. (2019) found that the PCR-RFLP of *hsp65* and multiposition sequencing based on ITS, *rpoB*, and 16S rRNA genes can be applied for NTM species identification [33]. It was reported that the sequence analysis based on the user base of 16s rRNA and *rpoB* was more dominant than that of ITS [33].

Among the NTM pulmonary diseases (PD), MAC infection is predominant, accounting for 60–80% of cases in the US and Japan and 50–75% of cases in Korea [19, 21, 22, 34]. The distribution of NTM may vary depending on the geographic region and environment. In Tehran, Iran, the main NTM species was *M*. *fortuitum* [33], whereas in another study, the main NTM species distributed in other regions of Iran was *M*. *kansasii* [35]. According to Ko et al. (2018), in a survey of the distribution of the major causative agents in patients with NTM infection at a tertiary referral hospital in Korea from 2001 to 2015, MAC was confirmed to be the major causative agent in 75% patients with NTM-PD for more than 15 years [34]. The number of NTM cases per 100,000 population showed a sharp increase in proportion compared to the increase in MAC cases, whereas the incidence of *M*. *abscessus* complex and *M*. *kansasii* remained relatively unchanged for 15 years [34]. Additionally, the proportion of *M*. *avium* over *M*. *intracellulare* in MAC has been increasing since 2005, and in MABC, the proportions of *M*. *abscessus* and *M*. *massiliense* were 54% and 46%, respectively [34]. Choi et al. (2013) identified 56 NTM strains isolated from patients in Busan-Gyeongnam, Korea, and found 15 (26.7%) strains of *M*. *intracellulare* type I, 14 (25%) *M*. *avium*, 11 (19.5%) *M*. *abscessus*, 3 (5.4%) *M*. *kansasii* type I, and 2 (3.6%) *M*. *pulveris* as well as lower amounts of *M*. *intracellulare* type 2, *M*. *chelonae*, *M*. *kansasii* type V, *M*. *gallinarum*, and *M*. *wolinskyi* [19]. In this study, using the mycobacterial diagnosis strategy, *M*. *intracellulare* (73.1%), *M*. *avium* subsp. *avium* (8.1%), *M*. *avium* subsp. *hominissuis* (4.1%), *M*. *fortuitum* (2.8%), *M*. *massiliense* (2.8%), *M*. *abscessus* (0.6%), *M*. *kansasii* (1.2%), and coinfections (2.2%) were found in patients with PD in Gyeongnam, Korea. Interestingly, *M*. *intracellulare* was found in 75% of cases, including the number of coinfection cases, making it the most dominant species in Gyeongnam, Korea. Moreover, it was confirmed that the proportion of MAC was significantly higher in NTM outbreaks in Korea, and more than 85.3% of MACs were distributed in the Gyeongnam region. The main cause of *M*. *avium* lung infection is contaminated domestic tap water, and *M*. *intracellulare* is believed to be found in environmental sources such as soil [34, 36]. Interestingly, the incidence of NTM was completely different according to the region and environment in Korea, and the incidence of NTM infection clearly reflects environmental characteristics [34, 37].

Additionally, the three clinical isolates (ND#1, ND#2, and ND#3), which were difficult to be identified by multigene sequence analysis, were predicted using a phylogenetic tree. The three clinical isolates showed close positions with MAC-related strains in the 16S rRNA and *hsp65* sequence-based tree analyses (Figures 3(a) and 3(c)). In particular, ND#2 showed high similarity to MAC in ITS; thus, ND#2 was assumed to be a MAC-related strain (Figure 3(d)). ND#1 and ND#3 with high similarity to each other showed similarity to the MTB complex in ITS, showing different characteristics from those of ND#2 (Figure 3(d)). Additionally, ND#1, ND#2, and ND#3 showed high similarity to each other in the *rpoB* sequence-based tree but showed completely separate groups from other NTM species (Figure 3(b)). Therefore, further analyses of the three samples are required.

## 5. Conclusions

In this study, a two-step mPCR was developed to identify NTM and MTB species in clinical isolates in Korea. This method quickly and accurately diagnosed major mycobacterial infection-causing species. In addition, other NTMs not identified by the two-step mPCR were identified by a novel multigene-sequencing method using gene targets, such as 16s rRNA, *rpoB*, *hsp65*, and ITS. Partial regions selected from these gene targets covering the entire gene sequence identified NTM species quickly and accurately. A total of 320 clinical isolates were identified. In the first mPCR, *M*. *intracellulare* was the most predominant species isolated in 234 (73.1%) cases, followed by *M*. *avium* subsp. *avium* (26, 8.1%) and *M*. *avium* subsp. *hominissuis* (13, 4.1%). In the second mPCR, *M*. *fortuitum* was isolated in 9 (2.8%), *M*. *massiliense* in 9 (2.8%), *M*. *abscessus* in 2 (0.6%), and *M*. *kansasii* in 4 (1.2%). Coinfection was also detected in 7 (2.2%) cases. This diagnostic strategy succeeded in the identification of 99.1% of clinical strains, particularly the identification of 95% of clinical strains, using only the two-step mPCR method. In addition, it was possible to diagnose coinfections due to major Mycobacterium species in patients. This study also showed the distribution of isolated NTM species in patients in the Gyeongnam region using clinically applicable diagnostic strategies. In the future, it will be necessary to further develop mPCR techniques for the faster diagnosis of clinical specimens. This diagnostic strategy using two-step mPCR and multigene sequence-based analysis can be very useful for the rapid and accurate identification of bacteria, resulting in reduced delays in treatment decisions.

## Figures and Tables

**Table 1:** Oligonucleotide primers used in the present study.

	Target gene	Species	Size (bp)	Oligonucleotide sequence (5′- 3′)	Ref
1^st^ mPCR	IS901	*M*. *avium* subsp. *avium*	753	F: GAACGCTGCTCTAAGGACCTGTTG	[21]
				R: GGAAGGGTGATTATCTGGCCTGC	
	rv0577	MTB complex	705	F: ATGCCCAAGAGAAGCGAATACA	[22]
				R: AATGTCAGCCGGTTCCGCAA	
	16S rRNA^*∗*^	All mycobacterial species	484	F: ATAAGCCTGGGAAACTGGGT	[21]
				R: CACGCTCACAGTTAAGCCGT	
	RD9	MTB	358	F: GTGTAGGTCAGCCCCATCC	[22]
				R: GTGTGGATTCCGTGGGCG	
	IS1311	*M*. *avium* complex	282	F: CATGAACGGAGCGCATCAC	In this study
				R: ATCGGCGCAAATCCGGGA	
	DT1	*M*. *intracellulare*	106	F: AAGGTGAGCCCAGCTTTGAACTCCA	[22]
				R: GCGCTTCATTCGCGATCATCAGGTG	

2^nd^ mPCR	Transferase	*M*. *kansasii*	582	F: ACTTCTTTCGTCGTCGCGAC	In this study
				R: GCAGAAGCACAGATCCCAACC	
	*mass_3210*	*M*. *abscessus*/*M*. *massiliense*	310/1145	F: GCTTGTTCCCGGTGCCACAC	[22]
				R: GGAGCGCGATGCGTCAGGAC	
	SOD	*M*. *fortuitum*	275	F: CCAAGCTCGATGAGGCGCGG	[27]
				R: CCGATCGCCCAGGCTGTCGT	

Sequencing	rpoB		950	F: TCGACGAGTGCAAAGACAAGGAC	In this study
				R: CTGGTGCCGAAGAACTCCTTGA	
	hsp65		640	F: AGGGTATGCGGTTCGACAAG	In this study
				R: GACGATGCCCTCCTCCACGG	
	ITS		420	F: GTGCGGCTGGATCACCTCCT	In this study
				R: GGTTGACAGCTCCCCGAGGCA	

MTB, *M*. *tuberculosis*; SOD, superoxide dismutase; rRNA, ribosomal RNA; ITS, internal transcribed spacer. ^*∗*^16S rRNA primers were used for the first mPCR and sequencing.

**Table 2:** Identification of NTM clinical isolates from patients with NTM pulmonary disease treated at Gyeongsang National University Hospital from 2016 to 2018.

	Clinical strains	No. of isolates	Percentage (%)
1	*M*. *intracellulare*	234	73.1
2	*M*. *avium* subsp. *avium*	26	8.1
3	*M*. *avium* subsp. *hominissuis*	13	4.1
4	*M*. *kansasii*	4	1.3
5	*M*. *massiliense*	9	2.8
6	*M*. *abscessus*	2	0.6
7	*M*. *fortuitum*	9	2.8
8	*M*. *chimaera*	6	1.9
9	*M*. *gordonae*	4	1.3
10	*M*. *colombiense*	1	0.3
11	*M*. *mageritense*	1	0.3
12	*M*. *persicum*	1	0.3
13	Coinfection^*∗*^	7	2.2
14	ND^*∗∗*^	3	0.9
	Total	320	100

^*∗*^The seven cases of coinfection included three cases of *M*. *avium* subsp. *avium* and *M*. *intracellulare* coinfection; one *M*. *avium* subsp. *hominissuis* and *M*. *intracellulare* coinfection; one *M*. *intracellulare* and *M*. *kansasii* coinfection; one MTB and *M*. *intracellulare* coinfection; and one MTB and *M*. *massiliense* coinfection.^*∗∗*^Three clinical isolates were not clearly identified by the first and second mPCR as well as multigene sequence-based analysis. ND, not determined.

**Table 3:** Results of multigene sequence-based analysis by comparing the four target gene sequences against the reference strains and those obtained from the NCBI database.

	16S rRNA		rpoB		hsp65		ITS	
Strains^*∗*^	Strains	Percent homology (%)	Strains	Percent homology (%)	Strains	Percent homology (%)	Strains	Percent homology (%)
CI-1	*M*. *chimaera* (CP023151.1)	100	*M*. *chimaera* (CP023151.1)	99	*M*. *chimaera* (CP023151.1)	100	*M*. *chimaera* (CP023151.1)	99.7
	*M*. *intracellulare* (CP023149.1)	100	*M*. *intracellulare* (CP003324.1)	96.7	*M*. *intracellulare* (CP003347.1)	100	*M*. *paraintracellulare* (AP022597.1)	99.7

CI-2	*M*. *gordonae* (MK630261.1)	99.8	*M*. *gordonae* (LQOY01000155.1)	99.9	*M*. *gordonae* (LQOY01000150.1)	99.7	*M*. *gordonae* (LR031451.1)	99.7
	*M*. *paragordonae* (MF437319.1)	99.8	*M*. *paragordonae* (CP025546.1)	96.1	*M*. *paragordonae* (CP025546.1)	95.3	*M*. *paragordonae* (CP025546.1)	98.3

CI-3	*M*. *gordonae* (MK630261.1)	100	*M*. *gordonae* (LQOY01000155.1)	100	*M*. *gordonae* (LQOY01000150.1)	98.1	*M*. *gordonae* (LR031451.1)	99.7
	*M*. *paragordonae* (MF437319.1)	100	*M*. *paragordonae* (CP025546.1)	96.2	*M*. *paragordonae* (CP025546.1)	93.9	*M*. *paragordonae* (CP025546.1)	98.3

CI-4	*M*. *colombiense* (CP020821.1)	100	*M*. *paraintracellulare* (AP022597.1)	97.1	*M*. *colombiense* (CP020821.1)	99	*M*. *colombiense* (CP020821.1)	98.4
	*M*. *intracellulare* (KM096752.1)	100	*M*. *colombiense* (CP020821.1)	96.6	*M*. *bouchedurhonense* (EU239790.1)	98.8	*M*. *mantenii* (AP022590.1)	96.8

CI-5	*M*. *chimaera* (CP023151.1)	99.3	*M*. *chimaera* (CP023151.1)	100	*M*. *chimaera* (CP023151.1)	100	*M*. *chimaera* (CP023151.1)	99.7
	*M*. *intracellulare* (CP023149.1)	99.3	*M*. *intracellulare* (CP003324.1)	97.7	*M*. *intracellulare* (CP003324.1)	100	*M*. *intracellulare* (CP023149.1)	99.7

CI-6	*M*. *chimaera* (CP045963.1)	100	*M*. *chimaera* (CP022223.1)	100	*M*. *chimaera* (CP045963.1)	99.8	*M*. *chimaera* (CP045963.1)	99.7
	*M*. *intracellulare* (KP736000.1)	100	*M*. *intracellulare* (CP003324.1)	97.5	*M*. *paraintracellulare* (AP022597.1)	99.8	*M*. *marseillense* (AP022584.1)	95.2

CI-7	*M*. *gordonae* (MK630261.1)	100	*M*. *gordonae* (LQOY01000155.1)	99.9	*M*. *gordonae* (LQOY01000150.1)	98.4	*M*. *paragordonae* (CP025546.1)	98.6
	*M*. *paragordonae* (MF437319.1)	100	*M*. *paragordonae* (CP025546.1)	96.3	*M*. *paragordonae* (CP025546.1)	95.7	*M*. *gordonae* (HG313849.1)	98.5

CI-8	*M*. *chimaera* (CP045963.1)	100	*M*. *chimaera* (CP022223.1)	100	*M*. *chimaera* (CP045963.1)	100	*M*. *chimaera* (CP045963.1)	100
	*M*. *intracellulare* (KP736000.1)	100	*M*. *intracellulare* (CP003324.1)	97.5	*M*. *paraintracellulare* (AP022597.1)	100	*M*. *marseillense* (AP022584.1)	95.4

CI-9	*M*. *persicum* (MN936127.1)	99.2	*M*. *kansasii* (KY933082.1)	100	*M*. *persicum* (MWKZ01000001.1)	99.1	*M*. *gordonae* (CP059165.1)	93.1
	*M*. *kansasii* (KP736051.1)	99.2	*M*. *persicum* (MWKZ01000001.1)	99.8	*M*. *kansasii* (CP006835.1)	97.2	*M*. *kansasii* (CP006835.1)	93.1
							*M*. *persicum* (MWKZ01000001.1)	90.9

CI-10	*M*. *mageritense* (AP022567.1)	99.6	*M*. *mageritense* (AP022567.1)	99.8	*M*. *mageritense* (AP022567.1)	97.6	*M*. *mageritense* (AP022567.1)	94
	*M*. *peregrinum* (AF537362.1)	99.6	*M*. *goodii* (AY262736.1)	96.1	*M*. *dioxanotrophicus* (CP020809.1)	97.6	*M*. *fortuitum* (KR995170.1)	87.6

CI-11	*M*. *marseillense* (AP022584.1)	99.8	*M*. *chimaera* (CP045963.1)	99.7	*M*. *chimaera* (CP045963.1)	99.8	*M*. *intracellulare* (CP003347.1)	100
	*M*. *intracellulare* (KR856204.1)	99.8	*M*. *intracellulare* (CP003324.1)	97.4	*M*. *paraintracellulare* (AP022597.1)	99.8	*M*. *marseillense* (AP022584.1)	98.6
	*M*. *chimaera* (CP045963.1)	99.5	*M*. *marseillense* (CP023147.1)	95	*M*. *timonense* (EU239792.1)	99.1	*M*. *chimaera* (CP023151.1)	98

CI-12	*M*. *gordonae* (MK630261.1)	100	*M*. *gordonae* (LQOY01000155.1)	100	*M*. *gordonae* (LQOY01000150.1)	100	*M*. *gordonae* (LR031451.1)	100
	*M*. *paragordonae* (MF437319.1)	100	*M*. *paragordonae* (CP025546.1)	96.2	*M*. *paragordonae* (CP025546.1)	95	*M*. *paragordonae* (CP025546.1)	98.7

CI-13	*M*. *chimaera* (CP022597.1)	100	*M*. *chimaera* (CP045963.1)	99.3	*M*. *chimaera* (CP023151.1)	99.5	*M*. *intracellulare* (CP003347.1)	99.5
	*M*. *colombiense* (CP020821.1)	100	*M*. *intracellulare* (CP003324.1)	97.1	*M*. *intracellulare* (CP003347.1)	99.5	*M*. *chimaera* (CP023151.1)	99

ND#1	*M*. *kansasii* (KF687952.1)	100	*M*. *kansasii* (KY933082.1)	95.2	*M*. *chimaera* (CP045963.1)	100	*M*. *basiliense* (LR130759.1)	93
	*M*. *riyadhense* (NR_044449.1)	100	*M*. *chimaera* (CP045963.1)	93.7	*M*. *paraintracellulare* (AP022597.1)	100	*M*. *marinum* (CP000854.1)	89.9
	*M*. *haemophilum* (MN535540.1)	99.7	*M*. *mantenii* (AP022590.1)	93	*M*. *timonense* (EU239792.1)	99.1	*M*. *kansasii* (CP019888.1)	89.6

ND#2	*M*. *marseillense* (AP022584.1)	100	*M*. *colombiense* (CP020821.1)	97.1	*M*. *colombiense* (CP020821.1)	97.9	*M*. *mantenii* (AP022590.1)	96.1
	*M*. *intracellulare* (KR856204.1)	100	*M*. *paraintracellulare* (AP022597.1)	96.6	*M*. *marseillense* (AP022584.1)	97.9	*M*. *chimaera* (CP022223.1)	96.1
	*M*. *colombiense* (CP020821.1)	99.7	*M*. *marseillense* (AP022584.1)	95.2	*M*. *chimaera* (CP023151.1)	97.6	*M*. *colombiense* (CP020821.1)	95.4

ND#3	*M*. *kansasii* (KF687952.1)	100	*M*. *kansasii* (KY933082.1)	95.2	*M*. *chimaera* (CP045963.1)	100	*M*. *basiliense* (LR130759.1)	92.6
	*M*. *riyadhense* (NR_044449.1)	100	*M*. *chimaera* (CP045963.1)	93.7	*M*. *paraintracellulare* (AP022597.1)	100	*M*. *marinum* (CP000854.1)	90
	*M*. *haemophilum* (MN535540.1)	99.7	*M*. *mantenii* (AP022590.1)	93	*M*. *timonense* (EU239792.1)	99.3	*M*. *shotsii* (AP022572.1)	89.3

^*∗*^Sixteen clinical isolates not determined by two-step mPCR were randomly numbered and named as CI-1–CI-13 and ND#1–ND#3.

## Data Availability

The data used to support the findings of this study are available from the corresponding author upon request.

## Supplementary Materials

Supplementary Materials Supplementary Figure 1: comparison of variation regions of ITS according to NTM species. Supplementary Table 1: sequence similarity values for full (upper right) and partial (lower left) 16S rRNA sequences of MAC reference strains. Supplementary Table 2: sequence similarity values for full (upper right) and partial (lower left) rpoB sequences of MAC reference strains. Supplementary Table 3: sequence similarity values for full (upper right) and partial (lower left) hsp65 sequences of MAC reference strains.
