# Supplementary material for: Identification of Nontuberculous Mycobacteria in Patients with Pulmonary Diseases in Gyeongnam, Korea, Using Multiplex PCR and Multigene Sequence-Based Analysis
Source: Can J Infect Dis Med Microbiol. 2021 Feb 22;2021:8844306. doi: 10.1155/2021/8844306 (PMC7920741; doi:10.1155/2021/8844306)
Supplement: Supplementary Materials — Supplementary Figure 1: comparison of variation regions of ITS according to NTM species. Supplementary Table 1: sequence similarity values for full (upper right) and partial (lower left) 16S rRNA sequences of MAC reference strains. Supplementary Table 2: sequence similarity values for full (upper right) and partial (lower left) rpoB sequences of MAC reference strains. Supplementary Table 3: sequence similarity values for full (upper right) and partial (lower left) hsp65 sequences of MAC reference strains. [file 8844306.f1.zip › 8844306.f1/Supplementary Figure.1.docx]

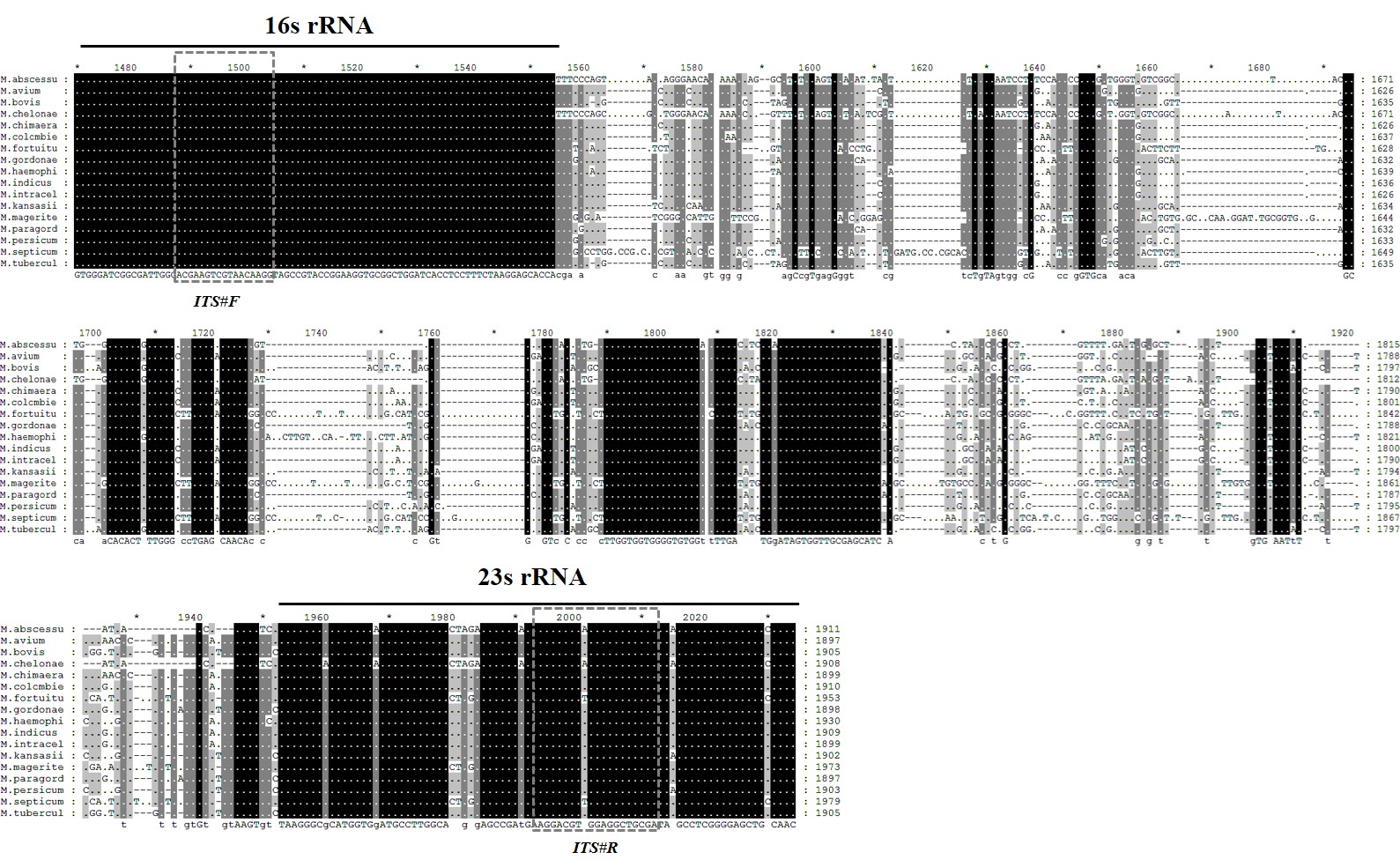
**Supplementary figure 1:** **Comparison of variation regions of ITS according to NTM species.** ITS regions of 17 reference strains (*M. abscessus* ATCC19977; *M. avium* 104; *M. bovis* BCG Pasteur 1173P2; *M. chelonae* ATCC19237; *M. chimaera* strain DSM 44623; *M. colombiense* CECT 3035; *M. fortuitum* ATCC6941; *M. gordonae* DSM44160; *M. haemophilum* DSM 44634; *M. intracellulare* ATCC 13950 ; *M. indicus* pranii MTCC 9506; *M. kansasii* ATCC 12478; *M. mageritense* CIP104973; *M. paragordonae* strain 49061; *M. septicum* strain ATCC 700731T; *M. tuberculosis* H37Ra ATCC25177; *M. xenopi* ATCC19250) were compared.
