# Supplementary material for: Identification of Nontuberculous Mycobacteria in Patients with Pulmonary Diseases in Gyeongnam, Korea, Using Multiplex PCR and Multigene Sequence-Based Analysis
Source: Can J Infect Dis Med Microbiol. 2021 Feb 22;2021:8844306. doi: 10.1155/2021/8844306 (PMC7920741; doi:10.1155/2021/8844306)
Supplement: Supplementary Materials — Supplementary Figure 1: comparison of variation regions of ITS according to NTM species. Supplementary Table 1: sequence similarity values for full (upper right) and partial (lower left) 16S rRNA sequences of MAC reference strains. Supplementary Table 2: sequence similarity values for full (upper right) and partial (lower left) rpoB sequences of MAC reference strains. Supplementary Table 3: sequence similarity values for full (upper right) and partial (lower left) hsp65 sequences of MAC reference strains. [file 8844306.f1.zip › 8844306.f1/Supplementary Table 3.docx]

Supplementary Table 3: Sequence similarity values for full (upper right) and partial (lower left) *hsp65* sequences of MAC reference strains

| **Species** | ***hsp65* partial (Lower left)/Full (Upper right) Sequence homology (%)** | | | | | | | | | | | | | | | | |
| --- | --- | --- | --- | --- | --- | --- | --- | --- | --- | --- | --- | --- | --- | --- | --- | --- | --- |
|  | **1** | **2** | **3** | **4** | **5** | **6** | **7** | **8** | **9** | **10** | **11** | **12** | **13** | **14** | **15** | **16** | **17** |
| **1. *M. abcessus*** ATCC19977 |  | 99.5 | 88.3 | 93.1 | 90.9 | 91.4 | 92.4 | 90.5 | 89.2 | 91.2 | 91.1 | 89.8 | 92.3 | 90.8 | 90.4 | 88.3 | 87.2 |
| **2. *M avium*** 104 | 91.7 |  | 90.9 | 89.7 | 89.7 | 96.6 | 91.4 | 93.0 | 92.6 | 95.7 | 95.7 | 92.2 | 91.3 | 93.4 | 90.9 | 90.9 | 89.9 |
| **3. *M. bovis*** BCG Pasteur 1173P2 | 90.1 | 91.9 |  | 87.8 | 90.6 | 90.6 | 88.3 | 89.8 | 91.1 | 90.6 | 90.6 | 90.8 | 88.3 | 90.2 | 91.1 | 100 | 89.4 |
| **4. *M. chelonae*** ATCC19237 | 95.5 | 91.7 | 89.1 |  | 89.0 | 90.3 | 91.4 | 88.5 | 88.5 | 89.4 | 89.3 | 89.7 | 91.5 | 90.0 | 89.7 | 87.4 | 86.9 |
| **5. *M. chimaera*** DSM 44623 | 93.2 | 95.6 | 91.9 | 91.4 |  | 97.2 | 91.3 | 92.7 | 92.1 | 99.1 | 99.2 | 91.6 | 90.8 | 93.2 | 91.9 | 90.6 | 89.5 |
| **6. *M. colombiense*** CECT 3035 | 92.5 | 95.9 | 91.4 | 92.2 | 97.9 |  | 92.3 | 92.9 | 92.3 | 97.2 | 97.2 | 91.8 | 91.7 | 93.8 | 92.3 | 90.6 | 89.2 |
| **7. *M. fortuitum*** ATCC6941 | 93.8 | 93.1 | 89.8 | 93.8 | 92.6 | 92.8 |  | 91.6 | 90.2 | 91.5 | 91.3 | 90.2 | 96.4 | 91.5 | 90.4 | 88.3 | 87.5 |
| **8. *M. gordonae*** DSM44160 | 92.6 | 93.1 | 90.4 | 92.5 | 93.2 | 92.7 | 93.1 |  | 91.9 | 92.6 | 92.5 | 91.6 | 91.0 | 96.6 | 91.9 | 89.8 | 89.3 |
| **9. *M. heamophilum*** DSM 44634 | 91.1 | 93.9 | 91.8 | 91.1 | 93.7 | 93.4 | 92.9 | 93.1 |  | 92.3 | 92.3 | 92.3 | 89.7 | 92.6 | 92.4 | 91.1 | 89.7 |
| **10. *M. indicus*** pranii MTCC 9506 | 93.4 | 95.5 | 91.8 | 91.6 | 99.6 | 97.7 | 92.8 | 93.4 | 93.6 |  | 99.8 | 91.4 | 91.1 | 93.5 | 91.9 | 90.6 | 89.6 |
| **11. *M. intracellulare*** ATCC 13950 | 93.2 | 95.6 | 91.9 | 91.4 | 100 | 97.9 | 92.6 | 93.2 | 93.7 | 99.6 |  | 91.4 | 91.0 | 93.4 | 91.9 | 90.6 | 89.7 |
| **12. *M. kansasii*** ATCC 12478 | 91.3 | 93.0 | 91.9 | 91.4 | 93.5 | 93.1 | 91.9 | 92.2 | 93.7 | 93.4 | 93.5 |  | 89.7 | 92.3 | 96.9 | 90.8 | 90.4 |
| **13. *M. mageritense*** CIP104973 | 93.2 | 92.5 | 89.9 | 93.6 | 92.5 | 92.3 | 96.8 | 92.9 | 92.9 | 92.6 | 92.5 | 91.0 |  | 90.7 | 90.4 | 88.3 | 87.3 |
| **14. *M. paragordonae*** 49061 | 92.5 | 92.9 | 90.8 | 91.7 | 94.3 | 93.8 | 92.8 | 95.9 | 93.7 | 94.4 | 94.3 | 93.3 | 92.2 |  | 92.6 | 90.2 | 89.5 |
| **15. *M. persicum*** 7MK | 91.8 | 92.5 | 89.9 | 93.8 | 92.5 | 92.3 | 96.8 | 92.9 | 94.6 | 92.6 | 92.5 | 97.6 | 91.8 | 93.7 |  | 91.1 | 90.4 |
| **16. *M. tuberculosis*** H37Ra ATCC25177 | 90.1 | 91.9 | 100 | 89.1 | 91.9 | 91.4 | 89.8 | 90.4 | 91.8 | 91.8 | 91.9 | 91.9 | 89.9 | 90.8 | 92.9 |  | 89.4 |
| **17. *M. xenopi*** ATCC19250 | 89.5 | 91.6 | 90.7 | 89.0 | 91.2 | 90.7 | 90.4 | 89.8 | 92.3 | 91.0 | 91.2 | 91.1 | 90.1 | 90.2 | 92.0 | 90.7 |  |
